# Hormonal regulation of cochlear gene expression: effects of the estrous cycle, ovariectomy, and estradiol treatment

**DOI:** 10.1186/s13293-026-00871-3

**Published:** 2026-03-08

**Authors:** Celia Zhang, Mengxiao Ye, Brandon Marzullo, Henry J. Adler, Bo Hua Hu

**Affiliations:** 1 Department of Audiology, School of Health Sciences, University of the Pacific, San Francisco, CA USA; 2 Department of Speech, Language, and Hearing, The University of Texas at Dallas, Dallas, TX USA; 3 UB Genomics and Bioinformatics Core NYS Center of Excellence in Bioinformatics & Life Sciences, University at Buffalo, Buffalo, NY USA; 4 Department of Computer Science and Engineering, University at Buffalo, Buffalo, NY USA

**Keywords:** Estrous cycle, Hormone regulation, Gene expression, Bioinformatics analyses, RNA sequencing, Cochlea, Mouse

## Abstract

**Background:**

The significance of sex differences in auditory function and vulnerability to hearing disorders has gained increasing attention. However, the underlying biological mechanisms remain unclear. Estrogen and other female hormones are known to regulate gene expression in various organs and tissues. However, their role in regulating gene expression in the cochlea remains to be determined. The current study investigated how female hormonal status, through natural fluctuations of the estrous cycle and hormonal deprivation via ovariectomy, modulates cochlear gene expression in mice.

**Methods:**

The cochlear transcriptome was examined under two experimental conditions that alter female hormones level: the normal estrous cycle and surgical removal of the ovaries (ovariectomy), with or without subsequent estradiol treatment. RNA sequencing was used to profile cochlear transcriptomes, followed by bioinformatic analyses to identify differentially expressed genes and their associated biological processes.

**Results:**

Our study identified a set of cochlear genes whose expression varies between the proestrus and diestrus stages of the estrous cycle. Most of these cochlear genes are autosomal protein-coding genes. There were more diestrus-biased genes compared to proestrus-biased genes. Many of these estrous cycle-regulated genes have been previously reported as hormone-responsive and encompass a range of functional categories, particularly those involved in regulating cellular function. The differentially expressed genes are primarily associated with immune-related functions. Notably, immune-related genes showed higher expression during diestrus. Our study also showed that ovariectomy altered cochlear gene expression, though it influenced only a limited number of genes. Ovariectomy-modulated genes were primarily associated with inflammatory responses and autophagy. Furthermore, several ovariectomy-induced changes were reversed by estradiol treatment, highlighting the regulatory role of this hormone in cochlear gene expression.

**Conclusions:**

Both natural hormonal fluctuations across the proestrus and diestrus stages of the estrous cycle, as well as experimental hormone manipulation through ovariectomy and estradiol treatment, can modulate cochlear gene expression. These findings suggest that hormone-driven transcriptional changes may contribute to sex differences in auditory physiology and disease vulnerability.

**Supplementary Information:**

The online version contains supplementary material available at 10.1186/s13293-026-00871-3.

## Abstract (plain language summary)

Sex differences in auditory function and susceptibility to hearing loss have been documented. One possible explanation involves the influence of female sex hormones on gene expression in the cochlea. This study examined the impact of hormonal changes on cochlear gene expression in female mice. In the first experiment, we investigated changes in gene expression across the natural estrous cycle. In the second, we removed the ovaries, the primary source of female hormones, to observe the effects of hormone loss on cochlear gene expression. Additionally, a subset of ovariectomized mice received estradiol to test whether estrogen treatment could reverse ovariectomy-induced changes. We identified a group of cochlear genes whose expression varied across the estrous cycle. Most of these genes were autosomal protein-coding genes. More genes were upregulated during the diestrus phase than during proestrus, and many of these genes were linked to immune-related functions. Notably, immune-related genes that showed higher activity during diestrus were not sex-biased. Ovariectomy also altered cochlear gene expression, though the number of affected genes was smaller than that observed across the estrous cycle. These genes were primarily associated with inflammation and autophagy. Some of these changes were reversed by estradiol treatment, highlighting the hormone’s role in regulating cochlear gene expression. Together, our findings demonstrate that natural hormone fluctuations across the proestrus and diestrus stages of the estrous cycle influence gene activity in the cochlea. Understanding hormonal regulation of cochlear gene expression provides new insights into sex-based differences in hearing and disease development.

**Supplementary Information:**

The online version contains supplementary material available at 10.1186/s13293-026-00871-3.

## Highlights

Cochlear gene expression varies in a stage-specific manner across the proestrus and diestrus stages of the estrous cycle in female mice.

Many of the differentially expressed genes are known targets of female sex hormone receptors.

Genes modulated by the estrous cycle are enriched for immune-related functions.

Most of these immune-related genes show higher expression during the diestrus stage.

Ovariectomy alters cochlear gene expression, although the number of affected genes is smaller than that observed across the estrous cycle.

**Supplementary Information:**

The online version contains supplementary material available at 10.1186/s13293-026-00871-3.

## Background

Sex is a biological variable that influences normal development, anatomy, physiological processes, and disease manifestation. This influence extends to the auditory system, where sex-related differences are evident in cochlear structure, function, as well as disease susceptibility and progression. Structurally, in both humans and mice, females generally have a shorter cochlear lengths [1] and greater efferent innervation to their inner hair cells [2], a type of sensory cells in the cochlea. Functionally, females demonstrate lower hearing thresholds at younger ages [3]. Furthermore, electrophysiological assessments reveal that females exhibit larger amplitudes and shorter latencies in auditory brainstem responses (ABRs) compared to males [4–6], reflecting enhanced neural activity in auditory signal transduction. Spontaneous otoacoustic emissions (SOAEs), which indicate normal outer hair cell function (another type of sensory cell in the cochlea), are more prevalent and exhibit greater amplitude in females than in males [7–10]. Females also show more robust distortion product otoacoustic emissions (DPOAEs), another measure of outer hair cell function, with higher amplitudes, and the enhancement of amplitude is frequency-dependent [11–15]. These observations demonstrate that sex exerts a significant impact on both structural and functional aspects of the auditory system.

Beyond anatomical and functional differences under normal conditions, the cochlea exhibits notable sex-based variations in susceptibility to cochlear pathogenesis and disease progression. Females typically experience a later onset of age-related hearing loss compared to males [16–18]. They also exhibit a delayed reduction in DPOAE amplitude compared to males [19]. Additionally, females are less susceptible to acoustic injury, as evidenced by smaller hearing threshold shifts [20]. Consistent with clinical observations, experimental studies in animals have also demonstrated reduced susceptibility to noise-induced threshold reduction in females [21, 22].

Despite growing evidence of sex differences in cochlear physiology and disease susceptibility, the underlying biological mechanisms remain poorly understood. Sex-dependent gene expression is a potential contributing factor to the observed sex differences in the auditory system. Our recent investigation identified distinct cochlear gene expression profiles in male and female mice [23]. Male-biased genes are predominantly associated with mitochondrial energy production and transcriptional regulation, whereas female-biased genes are linked to synaptic function. Notably, while some of these sex-biased genes are located on sex chromosomes, the majority are located on autosomes. Although these findings are essential for understanding the mechanisms underlying sex differences in cochlear function and disease development, the regulatory processes governing sex-biased gene expression in the cochlea remain unclear.

Hormonal influences, particularly those related to female sex hormones, represent another potential contributing factor to the observed sex differences. Studies have identified the presence of estrogen receptor subtypes in the cochlea, including estrogen receptor alpha (ERα) and beta (ERβ) [24]. These receptors have been implicated in protecting cochlear structures from acoustic injury [25]. Loss of their expression has been shown to impair hearing function [26]. Hearing sensitivity and ABR latencies are known to fluctuate across the menstrual cycle, correlating with variations in estrogen levels [27–32]. Similarly, DPOAE amplitudes vary across the menstrual cycle, with significantly higher values observed during the peri-ovulatory phase, coinciding with peak estrogen levels, compared to the luteal and follicular phases [33]. Moreover, menopause significantly accelerates hearing degeneration in older women, and serum estrogen levels are strongly correlated with hearing outcomes [34, 35]. Together, these findings suggest that female hormones play an essential role in modulating auditory function and protecting against cochlear degeneration.

Female hormones, particularly estrogen and progesterone, exert their biological effects primarily by regulating gene expression. Estrogens act via their receptors (ERα and ERβ), which translocate to the nucleus, bind to estrogen response elements (EREs) on DNA, and recruit co-activators or co-repressors to modulate gene transcription [36]. Progesterone operates similarly through progesterone receptors (PRs), influencing gene expression by binding to specific DNA sequences or modulating other transcription factor pathways, such as AP-1 and NF-κB [37]. Hormonal regulation is also context-dependent, affected by factors such as receptor subtype, chromatin structure, and the cellular microenvironment [38]. We hypothesize that hormone-mediated regulation of gene expression also occurs within the cochlea.

The present study was designed to investigate the effects of sex hormones on cochlear gene expression to better understand female hormone-responsive genes in the cochlea. We examined the cochlear transcriptome under two conditions that alter female hormone levels: the normal estrous cycle and surgical removal of the ovaries (ovariectomy, OVX). Our study identified a set of cochlear genes whose expression varies across the estrous cycle, with the majority being autosomal protein-coding genes. More genes were biased toward the diestrus phase than toward the proestrus phase. Many of these cycle-regulated genes are known to be hormone-responsive and span diverse functional categories, particularly those involved in regulating cellular functions. Immune-related genes were especially prominent, displaying higher expression during the diestrus phase. Additionally, our study revealed that OVX altered cochlear gene expression, although the number of affected genes was smaller. Several of the OVX-induced changes were reversed by estradiol treatment, underscoring estrogen’s regulatory role in cochlear gene expression. Together, our study reveals that female hormones influence cochlear gene expression, potentially contributing to sex-related differences in hearing and ear health.

## Methods

### Subjects

**Fig. 1:**
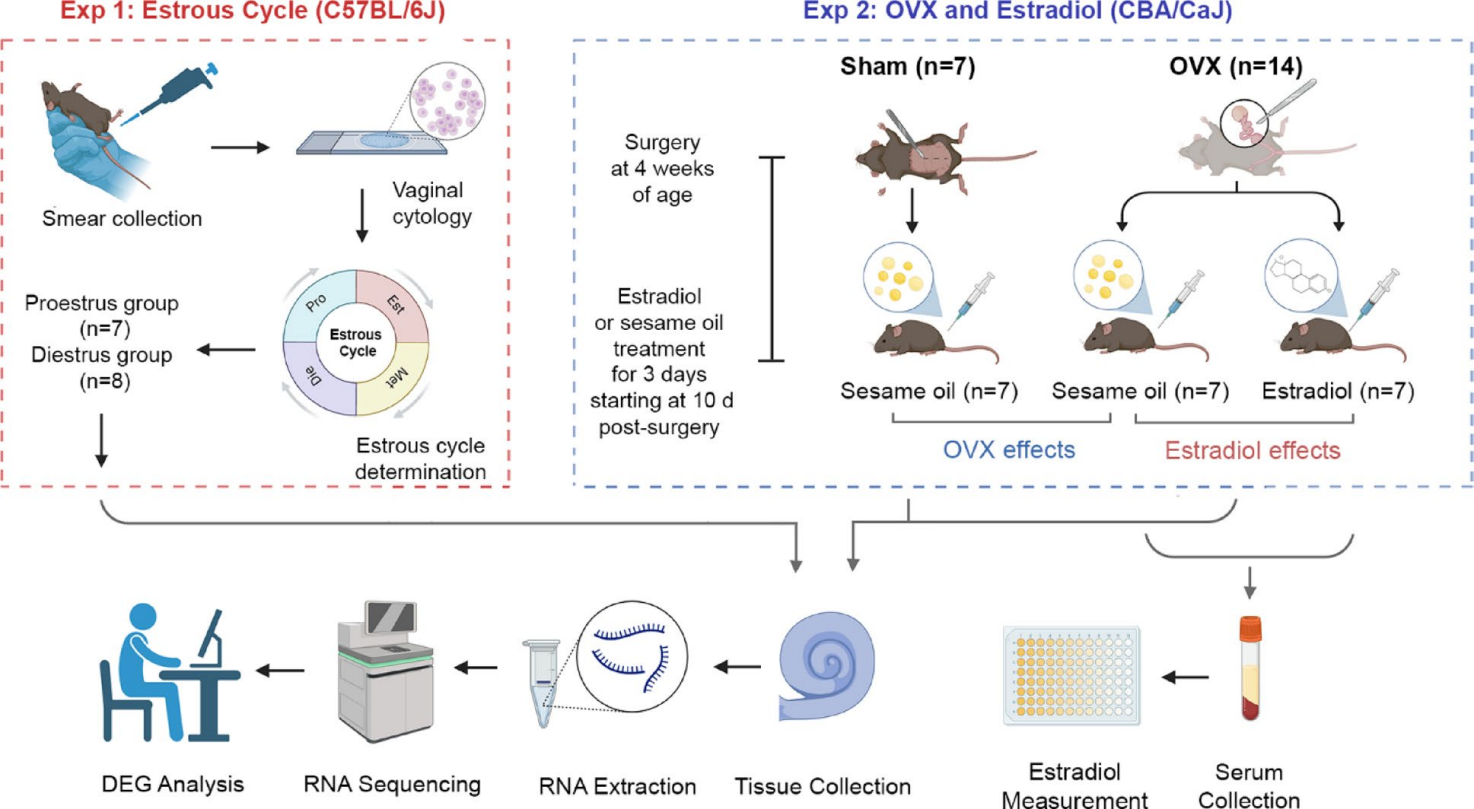
Schematics of experimental design and group assignments. The study included two experiments: Exp (1) cochlear transcriptome assessment during the normal estrous cycle, and Exp (2) cochlear transcriptome assessment after ovariectomy with or without subsequent estradiol treatment. For the first experiment, vaginal cytology was performed to determine the estrous cycle phase. Cochlear tissues were collected at the proestrus and diestrus phases for transcriptome analysis. For the second experiment, ovariectomy and sham surgery were performed. Ten days post-surgery, a group of ovariectomized mice received an injection of estradiol for three consecutive days. Another group of ovariectomized mice and a sham surgery control group received sesame oil treatment at the same timepoints. Cochlear tissues were then collected and prepared for transcriptome analysis. Blood samples were collected at the time of cochlear harvest to assess serum estradiol levels. OVX: Ovariectomy; Pro: Proestrus; Est: Estrus; Met: Metestrus; Die: Diestrus; DEG: Differentially Expressed Genes. Figure created by BioRender, with permission

Young, healthy female C57BL/6J and CBA/CaJ mice at the age of two months (The Jackson Laboratory, Bar Harbor, Maine, USA) were used in this study. Female mice were selected for this study, as the objective was to investigate the effects of female hormones. C57BL/6J mice were used in Experiment 1 (Fig. 1) to examine the impact of the estrous cycle on cochlear gene expression, as this strain was also used in our previous study on sex differences in cochlear gene expression [23], allowing data integration across studies. CBA/CaJ mice were used for Experiment 2 (Fig. 1) to investigate the effects of OVX and estradiol treatment. This strain was used because, unlike C57BL/6J mice, which carry a genetic mutation associated with early-onset hearing loss [39, 40], CBA/CaJ mice maintain normal hearing sensitivity throughout young and middle age [41]. All mice were housed under standard conditions (22 ± 1 °C, 12-hour light/dark cycle). All procedures were approved by the Institutional Animal Care and Use Committee at the State University of New York at Buffalo.

### Experimental design

The study included two experiments (Fig. 1). Experiment 1 examined the effects of the estrous cycle on cochlear gene expression. Fifteen female mice were included at the start of study and assigned, based on vaginal smear cytology, to two groups corresponding to different phases of the estrous cycle: proestrus (*n* = 7) and diestrus (*n* = 8). Then, the cochleae were harvested for RNA-seq analysis.

Experiment 2 investigated the effects of OVX and estradiol treatment on cochlear gene expression. A total of 21 mice were used, with 14 receiving OVX and 7 undergoing sham surgery. Cochlear tissues were collected from OVX mice (*n* = 7) and sham-operated controls (*n* = 7) to investigate the effect of ovarian hormone loss. An additional 7 OVX mice received estradiol treatment to determine whether estradiol could reverse OVX-induced changes in gene expression. Cochlear tissues from all groups were collected for RNA-seq analysis. Detailed experimental procedures and controls are described in the following sections.

### Estrous cycle determination

Vaginal cytology was used to determine the estrous cycle stage in female mice, as described in our previous work [23], with minor modifications to optimize the protocol. Briefly, mice were gently restrained, and approximately 15 µL of sterile saline was flushed through the vagina 2–3 times using a pipette. The final flush was collected and prepared as a wet smear on a glass slide. The procedure was performed daily between 9:00 and 10:00 a.m. for 7–14 days. To minimize stress, the procedure was completed within one minute by an experienced researcher. Mice exhibited reduced discomfort with repeated handling.

Smears were either left unstained or air-dried and stained with 0.1% crystal violet and then examined under a light microscope. Based on the predominant cell types, the estrous cycle was classified into four stages: diestrus, proestrus, estrus, and metestrus (see the Results section for representative images and classification criteria). For RNA-seq analysis, cochlear tissues were collected at either the proestrus or diestrus stage, as these two phases represent distinct hormonal states [42–45].

### Ovariectomy (OVX)

OVX was performed to induce ovarian hormone deprivation. The procedure was conducted at The Jackson Laboratory on female mice at the age of four weeks, following a protocol approved by the Jackson Laboratory Institutional Animal Care and Use Committee. Specifically, the surgery was performed on a HEPA-filtered, laminar-flow clean bench. Mice were anesthetized with isoflurane gas, and carprofen was administered for analgesia. Ophthalmic ointment was applied to protect the eyes, and body temperature was maintained using a controlled heat source.

The surgical site was prepared by shaving the fur and disinfecting the area with 70% ethanol and chlorhexidine. A skin incision was made parallel and ventral to the spine, followed by an incision through the abdominal wall to expose the left ovary, oviduct, and associated fat pad. These structures were removed using a crush-and-tear technique at the junction of the uterine horn and oviduct. The uterine horn was repositioned into the abdominal cavity, the abdominal wall was closed using absorbable sutures, bupivacaine was applied topically for local anesthesia, and the skin incision was closed with a wound clip. The same procedure was then repeated to remove the right ovary. A total of 14 mice received OVX.

Sham-operated control mice underwent the same surgical procedure, except that the ovary, oviduct, and associated fat pad were left intact. This group included seven mice and served as the control for the OVX group to assess the effects of OVX on cochlear gene expression. Following surgery, warm, sterile saline was administered subcutaneously, and the mice were placed in clean, warm cages under close observation until they were fully ambulatory. Mice were group-housed and monitored daily until they were shipped to our animal facility at 7 days post-surgery. Wound clips were removed before shipment.

### Estradiol treatment

To evaluate whether OVX-induced changes in cochlear gene expression could be reversed by estradiol replacement, 17β-estradiol (Millipore-Sigma, catalog # E2758) was administered to seven OVX mice. These mice received estradiol treatment starting 10 days post-surgery, which was the time that we received the mice from Jackson Lab. The hormone was initially dissolved in sesame oil (Millipore-Sigma, catalog # S3547) to prepare a 1 mg/mL stock solution, which was stored in a glass vial at 4 °C for no more than 4 days. Before each injection, a working solution of 17β-estradiol at 30 µg/mL was prepared by diluting the stock with sesame oil. Mice received a daily subcutaneous injection of 3 µg estradiol in 0.1 mL of oil, administered between 10:00 and 11:00 a.m. for three consecutive days. This dosage was selected based on previous studies showing that it achieves physiological estradiol levels in mouse models [46]. The remaining OVX mice (*n* = 7) received an equal volume (0.1 mL) of sesame oil without estradiol and served as the control group for estradiol treatment. It should be noted that the sham-operated mice also received sesame oil treatment, allowing them to serve as controls for assessing the effects of OVX, since the OVX mice received sesame oil as well.

### Serum estradiol measurement

Cardiac blood collection was performed immediately before cochlear tissue collection to obtain peripheral blood for serum hormone analysis in OVX mice treated with estradiol or sesame oil (control). Mice were deeply anesthetized with an intraperitoneal injection of ketamine (100 mg/kg) and xylazine (10 mg/kg). Once fully anesthetized, the thoracic cavity was opened to expose the heart. A sterile syringe with a 23-gauge needle was carefully inserted into the left ventricle, and blood was slowly drawn to minimize hemolysis. Typically, 0.5–1.0 ml of blood was collected per mouse. The collected blood was transferred into a 1.7 mL microcentrifuge tube and allowed to clot at room temperature for 90 min. Then, the clot was gently dislodged from the wall of the tube by running a wooden applicator stick along the inner surface of the tube. The sample was centrifuged at 2000 × g for 15 min at 4 °C. The serum was collected and stored at − 80 °C until further analysis.

The samples were shipped to the Ligand Assay and Analysis Core at the University of Virginia for measurement of serum estradiol using an ELISA kit (American Laboratory Products Co, catalog # 11-ESTHU-E01).

### Cochlear tissue collection

Mice were euthanized at the end of the experiment to collect cochlear tissues for RNA-seq analysis. Specifically, euthanasia was performed by decapitation under CO₂ gas anesthesia. The cochleae were rapidly extracted and immediately immersed in ice-cold Hank’s Balanced Salt Solution (Corning, catalog # 21-022-CV). The bony shell on the lateral side of the cochlea, facing the middle ear space, was quickly opened, and the cochlea was transferred into an RNA-stabilizing reagent (RNAlater; Qiagen, Valencia, CA, USA) to preserve RNA integrity. Then, microdissection was performed to collect cochlear tissues, including the lateral wall, sensory epithelium, and osseous spiral lamina. Tissues from both cochleae of each mouse were pooled to generate one sample. The isolated tissues were placed into RNase-free PCR tubes and stored at − 80 °C until RNA extraction.

### Total RNA extraction

Total RNA was extracted from cochlear tissues using the RNeasy Plus Micro Kit (Qiagen GmbH, catalog # 74034, Hilden, Germany), following the manufacturer’s instructions. Briefly, tissues were transferred into Buffer RLT Plus for cell lysis and homogenization. The lysate was then applied to a gDNA Eliminator spin column to remove genomic DNA. The flow-through was transferred to a RNeasy MinElute spin column, washed twice, and briefly dried. Finally, RNA was eluted in RNase-free water. The purified RNA samples were stored at − 80 °C until further gene expression analysis.

### Quality control of total RNAs for RNA-seq

Total RNA was isolated from mouse cochlear tissues and delivered to the Genomics and Bioinformatics Core at the University at Buffalo. Total RNA concentration was measured using the Qubit RNA High Sensitivity Assay Kit with a fluorometer (ThermoFisher Scientific, catalog # Q32855, Waltham, MA, USA). RNA integrity was assessed using the Agilent 5200 Fragment Analyzer System with the High Sensitivity RNA Kit (Agilent Technologies, catalog # DNF-472-0500, Santa Clara, CA, USA). Three samples from Exp. 1 showed either a low RNA Quality Number (RQN < 8) or RNA concentrations markedly different from other samples in the same group and were therefore excluded from subsequent RNA-seq analysis.

### Library preparation and sequencing

After quality control, 50 ng of total RNA was used as input to generate RNA libraries using the Illumina RiboZero Total Stranded RNA library prep kit with ribosomal RNA removal (Illumina, catalog # 20040529) as described in our previous publication [23]. Briefly, total RNA was hybridized with rRNA baits targeting mammalian (human/mouse/rat) rRNA. The baits were then removed using RNAClean XP beads (Beckman Coulter, catalog # A63987), and the remaining RNA was enzymatically fragmented to 100–200 bp. These RNA fragments were used as templates for first- and second-strand cDNA synthesis. Double-stranded cDNA fragments were trimmed, ligated to anchor adapters, cleaned with AMPure XP beads (Beckman Coulter, catalog # A63881), and PCR amplified using UDP barcodes (Illumina, catalog # 20040553). Following a second clean-up with AMPure XP Beads, RNA library quality was assessed using the Agilent Fragment Analyzer with the NGS high-sensitivity assay (Agilent, catalog # DNF-474-0500) and Qubit 3.0 High Sensitivity DNA assay. The final libraries were pooled, and the concentration of the pool was determined using the Quantabio Universal qPCR reaction kit (Quantabio, catalog #95210500) according to the manufacturer’s instructions. Pooled libraries were diluted to 1:20,000 in EB buffer (Qiagen, catalog # 19086) before qPCR quantification using a BioRad CFX96 and C1000 thermal cycler with the following parameters: one cycle at 95 °C for 2 min, 35 cycles at 95 °C for 5 s and 65 °C for 25 s, one cycle at 65 °C for 30 s, and 60 cycles starting at 65 °C and ramping up to 95 °C at a rate of 0.5 °C per second (for primer annealing assessment). Pooled libraries were then diluted to 1.25 nM using EB buffer (Qiagen, catalog #19086) and denatured using 0.2 N NaOH for 8 min. PhiX (0.25 nM) was added before denaturation as a sequencing control (final concentration at 1%). The pooled library was loaded onto a NovaSeq6000 SP flow cell (PE100) for paired-end sequencing at a final concentration of 250 pM.

### RNA-seq raw data processing and differential expression analysis

Per-cycle basecall (BCL) files generated by the Illumina NovaSeq 6000 were converted to per-read FASTQ files using bcl2fastq2 version 2.20.0 using default parameters. The quality of the sequencing was reviewed using FastQC version 0.11.9 [47]. Detection of potential contamination was done using FastQ Screen version 0.14.0 [48]. FastQC and FastQ Screen quality reports were summarized using MultiQC version 1.14 [49].

Transcript quantifications were performed using Salmon version 1.10.1 [50], with mapping validation, sequence bias, and gc bias parameters enabled. The salmon index used was created using the genome and transcriptome references from the Gencode Mouse transcriptome, release 35. Salmon quality statistics were summarized using MultiQC.

Transcript quantifications were summarized to the gene level using tximeta version 1.24.0 [51]. Differentially expressed genes were detected using the Bioconductor package DESeq2 version 1.46.0 [52]. DESeq2 tests for differential expression using a negative binomial generalized linear model, dispersion estimates, and logarithmic fold changes. DESeq2 calculates log_2_ fold changes and Wald test *p*-values, as well as performing independent filtering and adjusting for multiple testing using the Benjamini-Hochberg procedure to control the false discovery rate (FDR). Log_2_ fold changes were shrunken using Apeglm version 1.28.0 [53].

### Functional enrichment analysis

Five online bioinformatics tools were used to examine the functional significance of differentially expressed genes, which were defined by an adjusted *p*-value < 0.05 and an absolute log₂ fold change > 0.2. First, we analyzed these genes using the Database for Annotation, Visualization, and Integrated Discovery (DAVID) [54]. The gene list was submitted to DAVID for functional annotation, and enriched biological processes and cellular components were identified based on Benjamini-adjusted *p*-values of 0.05, calculated using the linear step-up method [55]. For the DAVID analysis that yielded no significant findings with the Benjamini-adjusted *p*-value, we instead used the uncorrected *p*-value threshold (*p* < 0.05) to identify associated biological processes. This allowed us to capture potential trends that might otherwise be overlooked under more stringent correction. Second, we applied Gene Set Enrichment Analysis (GSEA) to assess the functional implications of the differentially expressed genes. Unlike DAVID, which only analyzes genes with significant fold changes, GSEA considers the entire ranked list of genes, regardless of their p-values or fold changes [56, 57]. For this analysis, we used the Hallmark gene set collection from MSigDB, which includes 50 curated gene sets that represent major biological pathways, such as inflammatory response, apoptosis, complement activation, estrogen response, and oxidative phosphorylation [58]. Gene sets were considered enriched if they met a normalized enrichment score threshold with a Family-Wise Error Rate (FWER) q-value < 0.01. Third, we used the PANTHER Classification System (Version 19.0), a web-based platform for exploring gene/protein classes, subfamilies, orthologs, paralogs, molecular functions, and pathways [59, 60]. The Gene List Analysis module was used to classify differentially expressed genes identified in our study, with the “Functional Classification Viewed in Gene List” option selected to generate detailed annotations. Fourth, we performed pathway enrichment analysis using the Kyoto Encyclopedia of Genes and Genomes (KEGG) database to identify molecular pathways associated with differentially expressed genes. Finally, TFLink (Transcription Factor Link) [61] was used to investigate transcriptional regulation. TFLink provides high-quality information on transcription factors, including their target genes, binding site sequences, and genomic locations. We used this tool to identify target genes of female hormone receptors.

### Data visualization and statistical analysis

Principal Component Analysis (PCA), a commonly used method for visualizing gene expression patterns, was performed to assess clustering of the expression data [62]. The PCA plot was generated using the ggplot2 package [63] in R version 4.3.

Statistical analyses were conducted using OriginPro (OriginLab, Northampton, MA, USA) or SigmaPlot (San Jose, version 10.0.1.25, CA, USA). Normality tests and equal variance tests were performed prior to statistical analysis. Student’s t-test was used when these assumptions were satisfied; otherwise, appropriate non-parametric tests were applied. An α-level of 0.05 was chosen to denote significance for all statistical tests. All data are presented as mean ± 1 standard deviation. Sample sizes were calculated using G*Power (version 3.1.9.2) when similar data from previous studies were available, aiming for 80% power to detect biologically meaningful differences. When such data were not available, sample sizes were determined using pilot observations. If the estimated sample size exceeded experimental feasibility, a reduced sample size was used to allow for the detection of trends or preliminary differences.

## Results

In the current study, we investigated the effects of female hormones on cochlear gene expression under two experimental conditions: natural hormonal fluctuations across the estrous cycle and hormone deficiency induced by ovariectomy (OVX). The results are organized into two sections, each dedicated to one condition.

### Experiment 1. Estrous cycle-dependent variations in cochlear gene expression

**Fig. 2:**
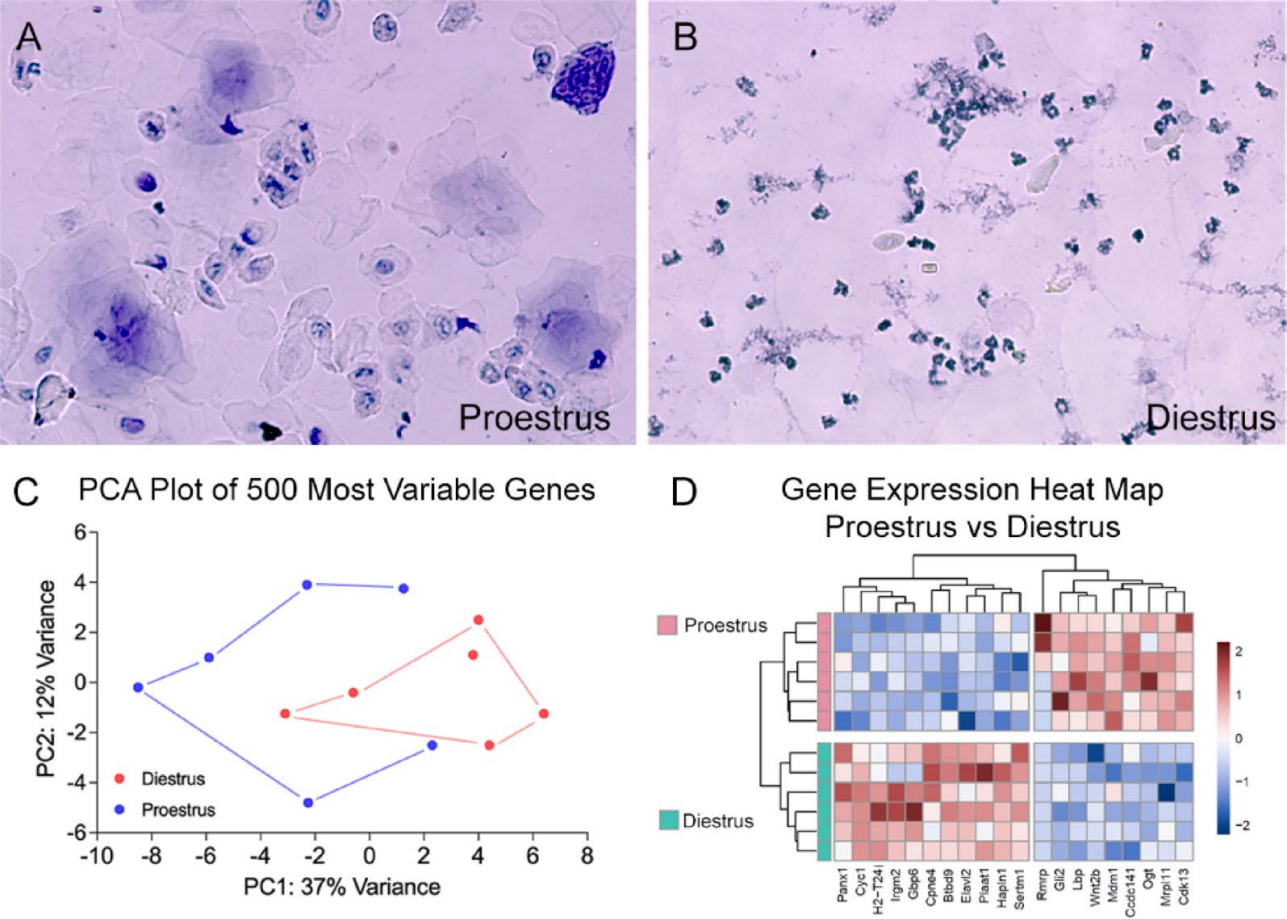
Estrous cycle identification and principal component analysis of gene expression variation across the estrous cycle. (**A**) Vaginal smear cytology during the proestrus stage, showing mostly round or oval nucleated epithelial cells, which are a hallmark of this phase. (**B**) Vaginal smear cytology during the diestrus stage, showing many small leukocytes with only a few epithelial cells, a pattern that clearly distinguishes this phase from proestrus. (**C**) Principal Component Analysis (PCA) of the top 500 variable genes in the diestrus and proestrus samples. The x-axis and y-axis represent the two principal components (PC1 and PC2). PC1 explains 37% of the total variance, whereas PC2 accounts for 12% of the total variance. While some overlap exists, many diestrus samples (red circles) are separate from proestrus samples (blue circles), suggesting phase-specific gene expression differences. (**D**). Heatmap of RNA-seq gene expression differences between proestrus and diestrus samples. Hierarchical clustering was performed on both genes and samples. The color scale represents relative expression levels, with dark red indicating higher expression and dark blue indicating lower expression

To investigate how the estrous cycle affects cochlear gene expression in mice, we analyzed transcriptomes from two stages of the cycle: proestrus and diestrus, as these stages represent distinct hormonal states. Estrous cycle stages were identified based on the cellular composition of the vaginal smears as described in previous publications [64, 65], including our prior work [23]. The proestrus stage was marked by a predominance of round to oval nucleated epithelial cells (Fig. 2A). In contrast, the diestrus stage showed abundant small leukocytes and only a few epithelial cells (Fig. 2B). Cochleae were then collected from mice in these two phases for transcriptome analysis. After excluding samples with poor RNA quality during quality control, 12 samples were included in the final data set: six from proestrus and six from diestrus.

To assess whether these samples could be distinguished based on gene expression variability, we performed a PCA of the top 500 most variable genes. As shown in Fig. 2C, Principal Component 1 (PC1) accounts for 37% of the variance, while Principal Component 2 (PC2) explains 12%. Proestrus and diestrus samples appear to separate along both components, suggesting possible stage-specific gene expression patterns. Figure 2D shows a heatmap displaying RNA-seq gene expression differences between proestrus and diestrus samples, with hierarchical clustering applied to both genes and samples. The transcriptomic profile during proestrus is distinctly different from diestrus, with clear bidirectional gene regulation and strong clustering by estrous stage. Overall, these analyses indicate that the estrous cycle is a biological factor that influences cochlear gene expression.

#### Differential expression of cochlear genes between the proestrus and diestrus phases

Our RNA-seq analysis detected over 20,000 genes in the cochlea. Differentially expressed genes between the proestrus and diestrus phases were identified using an adjusted *p*-value < 0.05 and an absolute log_2_ fold change > 0.2. This analysis identified 51 differentially expressed genes, which account for a small portion of the genes detected in the cochlea (Supplementary Table 1). This result suggests that although the estrous cycle influences cochlear gene expression, its impact is limited to a relatively small subset of genes.

**Fig. 3:**
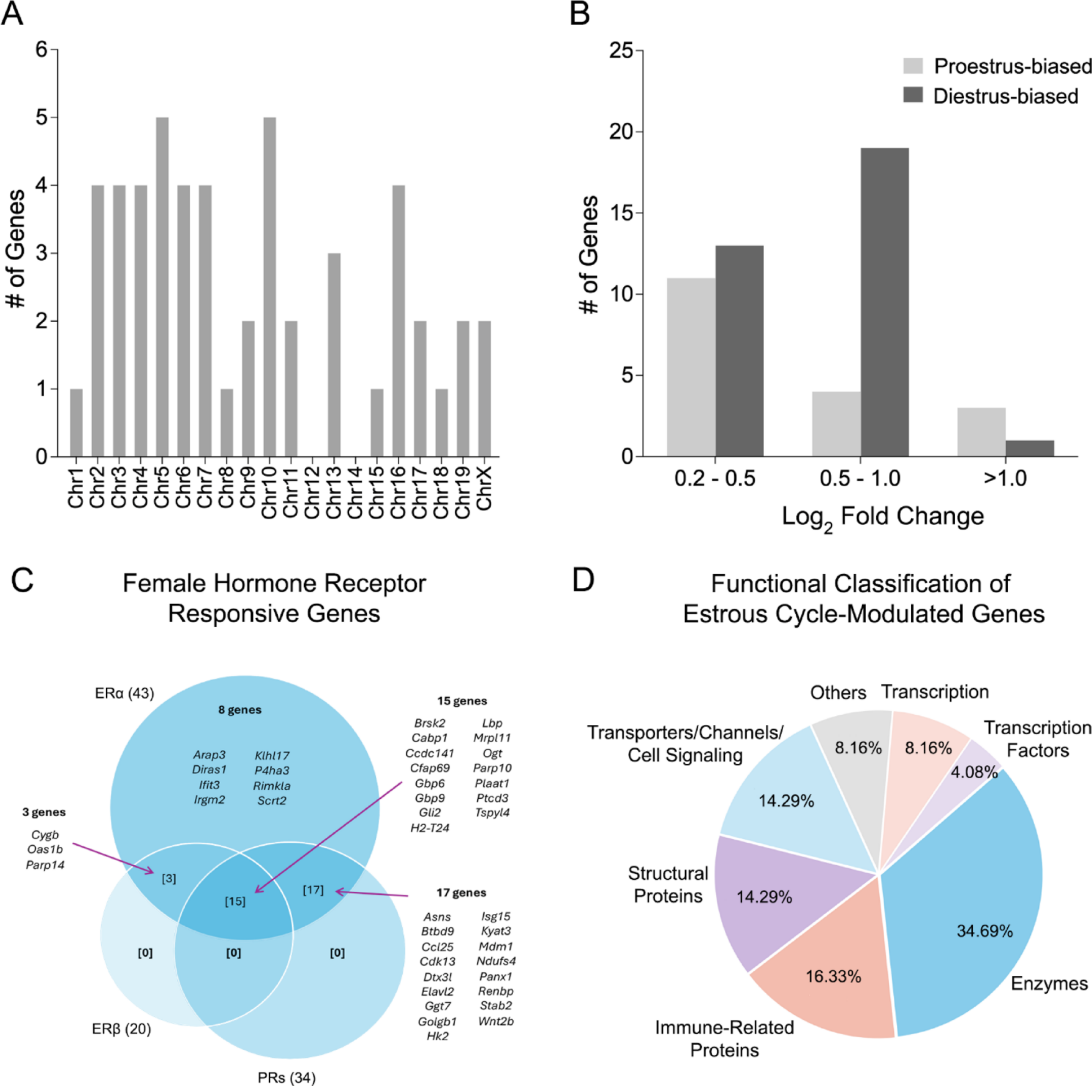
Characteristics and functional annotation of genes differentially expressed between the proestrus and diestrus phases. (**A**) Chromosomal distribution of differentially expressed genes. (**B**) Distribution of differentially expressed genes by log₂ fold change, showing that the majority fall between 0.2 and 1.0. (**C**) Hormone receptor responsiveness of estrous cycle–regulated genes. Among the 51 responsive genes, 43 are targets of ERα, 20 are targets of ERβ, and 34 are targets of PRs, with overlapping targets among receptors. (**D**) Functional classification of proteins encoded by the differentially expressed genes, with enzymes representing the largest category, followed by immune-related and structural proteins

All but one of differentially expressed genes were protein-coding; the sole exception was Gm10575, a ncRNA. These genes were distributed across all autosomes except chromosomes 12 and 14, and on the X chromosome (Fig. 3A). As expected, none were found on the Y chromosome, since all mice used in the study were female.

Among the 51 differentially expressed genes, the majority (33 genes) were upregulated in the diestrus phase (diestrus-biased), while 18 genes exhibited higher expression during the proestrus phase (proestrus-biased). This pattern of diestrus-dominant expression bias has also been observed in brain tissues [66]. Expression differences were generally modest, with log_2_ fold changes ranging from 0.2 to 1.0 for most genes (Fig. 3B). The greatest log_2_ fold change was observed for *Cfap69* (log_2_ fold change = 1.4, proestrus-biased), which encodes CFAP69, an important contributor to the structure and function of cilia, hair-like organelles found on the surface of many cells such as spermatozoa [67, 68] and olfactory receptors [69]. However, to our knowledge, *Cfap69* and its encoded protein CFAP69 have not previously been associated with inner ear function.

#### Target genes of sex hormone receptors

To determine whether the 51 estrous cycle–regulated genes could be regulated by female sex hormones, we examined their overlap with known targets of female hormone receptors. Using TFLink, an online bioinformatics tool, we retrieved known target genes of three major female hormone receptors: two estrogen receptors (ERα and ERβ) and one progesterone receptor (PR), and we compared them with our data set. This analysis revealed 43 overlapped genes, accounting for 84.3% of estrous cycle–regulated genes (43 of 51). Of these, 43, 20, and 34 genes were linked to the ERα, ERβ, and PRs, respectively (Fig. 3C). Notably, all ERα-responsive genes also responded to ERβ and PRs. Overall, this analysis revealed that the majority of estrous cycle–regulated genes in the cochlea are potential targets of estrogen and progesterone receptors.

#### Functional implications of estrous cycle-modulated genes

To explore the biological significance of the differentially expressed genes, we utilized the PANTHER Classification System (Version 19.0) to identify the functional categories of the proteins encoded by the genes identified in this study as being regulated across the estrous cycle. Identified gene functions were further manually curated to refine the classification. As shown in Fig. 3D, enzymes formed the largest category (34.69%), followed by immune-related proteins (16.33%), structural proteins (14.29%), and cell signaling molecules (14.29%). The detailed gene lists corresponding to each protein class are provided in Supplementary Table 2. This analysis suggests that cellular regulation is a primary function associated with estrous cycle-regulated genes.

We then conducted functional enrichment analyses using the Database for Annotation, Visualization, and Integrated Discovery (DAVID). Using a Benjamini-adjusted *p*-value threshold of < 0.05, we identified distinct biological processes associated with diestrus-biased and proestrus-biased genes. Diestrus-biased genes were primarily involved in immune processes, including innate immunity, general immunity, and antiviral defense (Table 1). The gene lists associated with each biological process are provided in Supplementary Table 3. Cellular component and molecular function analyses failed to yield any significant results. Gene ontology (GO) analysis further confirmed these findings by identifying enriched immune response-related terms, including innate immune response, cellular response to interferon-beta, immune system process, defense response to protozoan, adhesion of symbiont to host, cellular response to type II interferon, and defense response to bacterium (Table 1 and Supplementary Table 3). In contrast, proestrus-biased genes did not reveal enrichment in any biological processes or cellular components (data not shown). Together, these observations indicate that diestrus-biased genes play a prominent role in immune regulation.

**Table 1:** Biological processes and GO terms associated with diestrus-biased genes

Biological Processes	Benjamini- *p*-value	Gene Count
***Functional Annotation***		
Innate immunity	0.00013	8
Immunity	0.00603	9
Antiviral defense	0.00844	4
***GO Terms for Biological Processes***		
Innate immune response	0.00001	10
Cellular response to interferon-beta	0.00021	5
Immune system process	0.00078	9
Defense response to protozoan	0.00136	4
Adhesion of symbiont to host	0.00270	3
Cellular response to type II interferon	0.01070	4
Defense response to bacterium	0.01070	5

To further explore the functional implications of differentially expressed genes, we conducted Gene Set Enrichment Analysis (GSEA), a computational method that offers a broader view of functional associations. Unlike DAVID, which analyzes only differentially expressed genes, GSEA assesses the entire gene expression dataset. For this analysis, we utilized the Hallmark gene set collection from MSigDB, which comprises 50 curated gene sets representing well-defined biological processes and states, including inflammatory response, apoptosis, complement activation, estrogen response, and oxidative phosphorylation [58]. The FWER (Family-wise error rate) *p*-value of < 0.05, which offers a conservative measure of significance, was applied to identify significantly enriched gene sets. For the diestrus-biased genes, the two most strongly enriched sets were those involved in interferon-alpha and interferon-gamma responses, both of which met the significance threshold (Table 2). These findings are consistent with the DAVID analysis, reinforcing the finding that the diestrus phase is characterized by elevated immune activity. In contrast, no gene sets showed significant enrichment among proestrus-biased genes (data not shown). Collectively, DAVID and GSEA results highlight immune function as the primary biological process modulated by the estrous cycle-modulated genes in the cochlea.

**Table 2:** Gene sets associated with diestrus-biased genes identified by GSEA

Gene Set	NES	FWER q-value
Interferon alpha response	0.64	0.000
Interferon gamma response	0.54	0.000

NES = normalized enrichment score; FWER = Family-Wise Error Rate

#### Association of differentially expressed genes with the NOD-like receptor signaling pathway

**Fig. 4:**
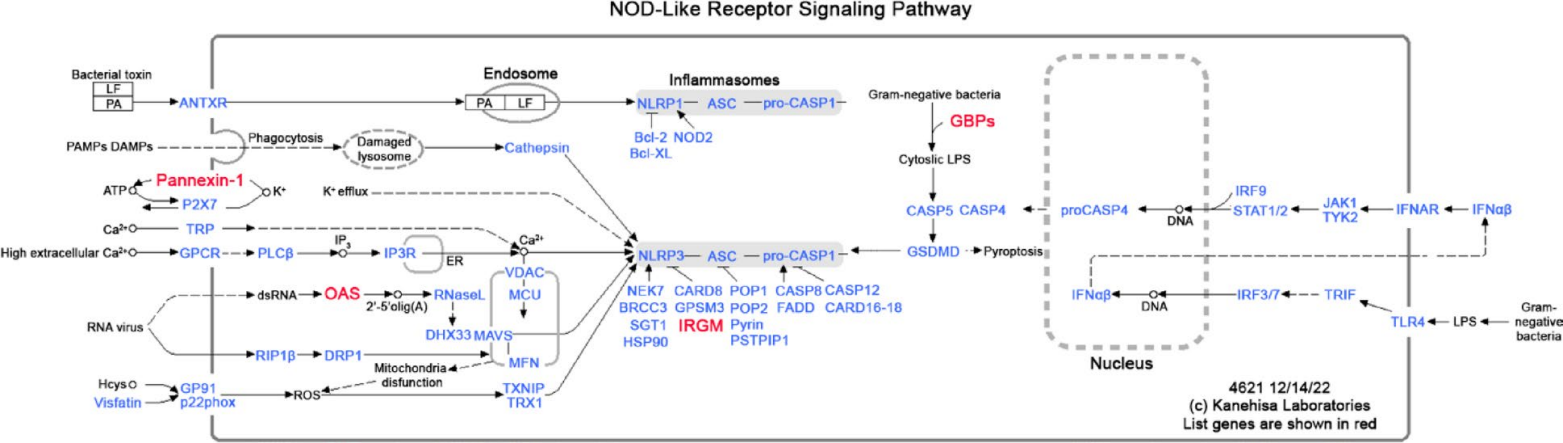
NOD-like receptor signaling pathway identified via KEGG pathway analysis of differentially expressed genes. Genes highlighted in red represent the estrous cycle-regulated genes identified in the current study. The figure was obtained from the KEGG website and simplified to enhance visibility

To identify molecular pathways associated with differentially expressed genes, we performed pathway enrichment analysis using the Kyoto Encyclopedia of Genes and Genomes (KEGG) database. Analysis of all 51 differentially expressed genes revealed the NOD-like receptor signaling pathway as the top-enriched pathway (Fig. 4), with a *p*-value of 0.012. This pathway comprises four genes identified in the current study as being modulated by the estrous cycle: 2’-5’ oligoadenylate synthetase 1B (*Oas1b*), guanylate-binding protein 3 (*Gbp3*), immunity-related GTPase family M member 2 (*Irgm2*), and pannexin-1 (*Panx1*). This pathway is a key component of the innate immune system, responsible for detecting intracellular pathogens and cellular stress, and initiating inflammatory responses [70, 71]. When the analysis was restricted to diestrus-biased genes, the NOD-like receptor signaling pathway not only remained the top-ranked pathway but also showed stronger significance (*p* = 0.0021). Notably, all four contributing genes were upregulated during diestrus, indicating that the enrichment is primarily driven by the genes with diestrus-based expression.

In addition to the NOD-like receptor signaling pathway, three other pathways were enriched, including metabolic pathways, amino sugar and nucleotide sugar metabolism, and alanine, aspartate, and glutamate metabolism (Supplementary Table 4). However, none of these pathways reached statistical significance (*p* > 0.05).

#### Overlap of the estrous cycle-regulated genes and sex-biased genes

Our previous study identified a set of sex-biased genes in the mouse cochlea [23], prompting us to hypothesize that some of these sex-biased genes may also be regulated by the estrous cycle. To investigate this, we incorporated RNA-seq data from that study into our current analysis. To ensure consistency in identifying differentially expressed genes, we re-analyzed the earlier dataset using the same bioinformatic pipeline applied in the current study. We also used the same criteria, an adjusted *p*-value < 0.05 and a log_2_ fold change > 0.2, to define differentially expressed genes. This analysis revealed a total of 3,370 sex-biased genes (1,804 male-biased and 1,566 female-biased).

**Fig. 5:**
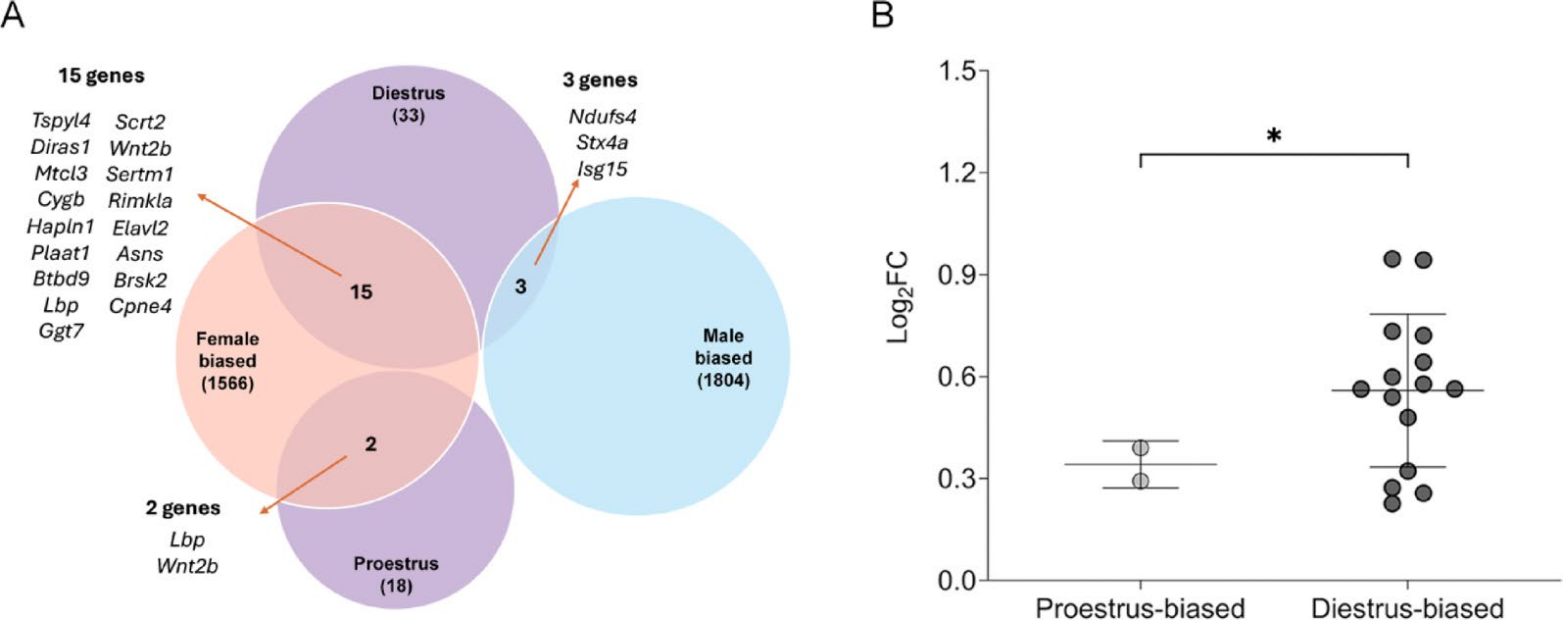
Overlap between sex-biased genes and estrous cycle-modulated genes. (**A**) Overlap between sex-biased genes (male- and female-biased) and estrous stage-biased genes (proestrus- and diestrus-biased). A total of 20 genes are identified as both sex-biased and estrous cycle-biased. Of these, 17 are female-biased and only three are male-biased. Among the 17 female-biased, estrous cycle-modulated genes, 15 are diestrus-biased. (**B**) Diestrus-biased genes generally exhibit larger log_2_ fold changes. On average, these genes show greater expression differences than proestrus-biased genes. * indicates *p* < 0.05

We next examined the overlap between these sex-biased genes and the 51 estrous cycle-modulated genes identified in the current study. This analysis revealed 20 overlapping genes (Fig. 5A), the majority of which were female-biased (17 out of 20). Notably, most of these female-biased genes were also diestrus-biased (15 out of 17). Most diestrus-biased genes exhibited a greater log_2_ fold change and their mean expression differences were significantly greater than those of proestrus-biased genes (Fig. 5B, two-tailed unpaired Student’s *t*-test with Welch’s correction, t = 2.86, df = 5.09, *p* = 0.034). This finding aligned with our earlier observation that diestrus-biased genes exhibit greater expression changes than proestrus-biased genes. Collectively, these results indicated that a subset of female-biased cochlear genes is also modulated by the estrous cycle, with elevated expression predominantly during the diestrus phase.

#### Different functional implications for sex-biased and non-sex-biased estrous cycle-modulated genes

As discussed above, our analysis identified two groups of estrous cycle-modulated genes: those with sex-biased expression and those without. To explore whether these groups have distinct functional implications, we conducted separate DAVID analyses for each group and uncovered striking differences in their functional profiles. As shown in Table 3 (also Supplementary Table 3), estrous cycle-modulated genes without sex-biased expression were significantly enriched for immune-related functions. The identified biological processes were innate immunity and general immune function, both with a Benjamini-adjusted *p*-value < 0.05. Consistently, GO term analysis revealed enrichment of multiple immune-related categories. In contrast, no significant biological processes were associated with genes that were both estrous cycle-modulated and sex-biased. Notably, 8 out of 9 estrous cycle-modulated genes contributing to immune-related functions were diestrus-biased. These findings demonstrate that the strong association with immune-related functions is primarily driven by diestrus-biased genes that do not exhibit sex-biased expression.

**Table 3:** Biological processes and GO terms associated with estrous cycle-modulated genes that do not exhibit sex-biased expression

Biological Processes	Benjamini *p*-value	Gene Count
***Functional Annotation***		
Innate immunity	0.00007	8
Immunity	0.00328	9
***GO Terms for Biological Processes***		
Immune system process	0.00010	10
Cellular response to interferon-beta	0.00017	5
Innate immune response	0.00042	8
Defense response to protozoan	0.00123	4
Adhesion of symbiont to host	0.00270	3
Defense response to bacterium	0.01030	5
Defense response to Gram-positive bacterium	0.04980	4

### Experiment 2. Effects of Ovariectomy (OVX) on Cochlear Gene Expression

To further investigate the regulation of cochlear gene expression by female hormones, we performed OVX to induce ovarian hormone deprivation. Sham-operated mice served as controls. To minimize estrous cycle variability in the control group, cochlear tissues were collected during the proestrus phase. By comparing gene expression profiles between OVX and sham mice, we evaluated the impact of hormone deficiency on cochlear gene expression.

#### OVX-induced expression changes of cochlear genes

**Fig. 6:**
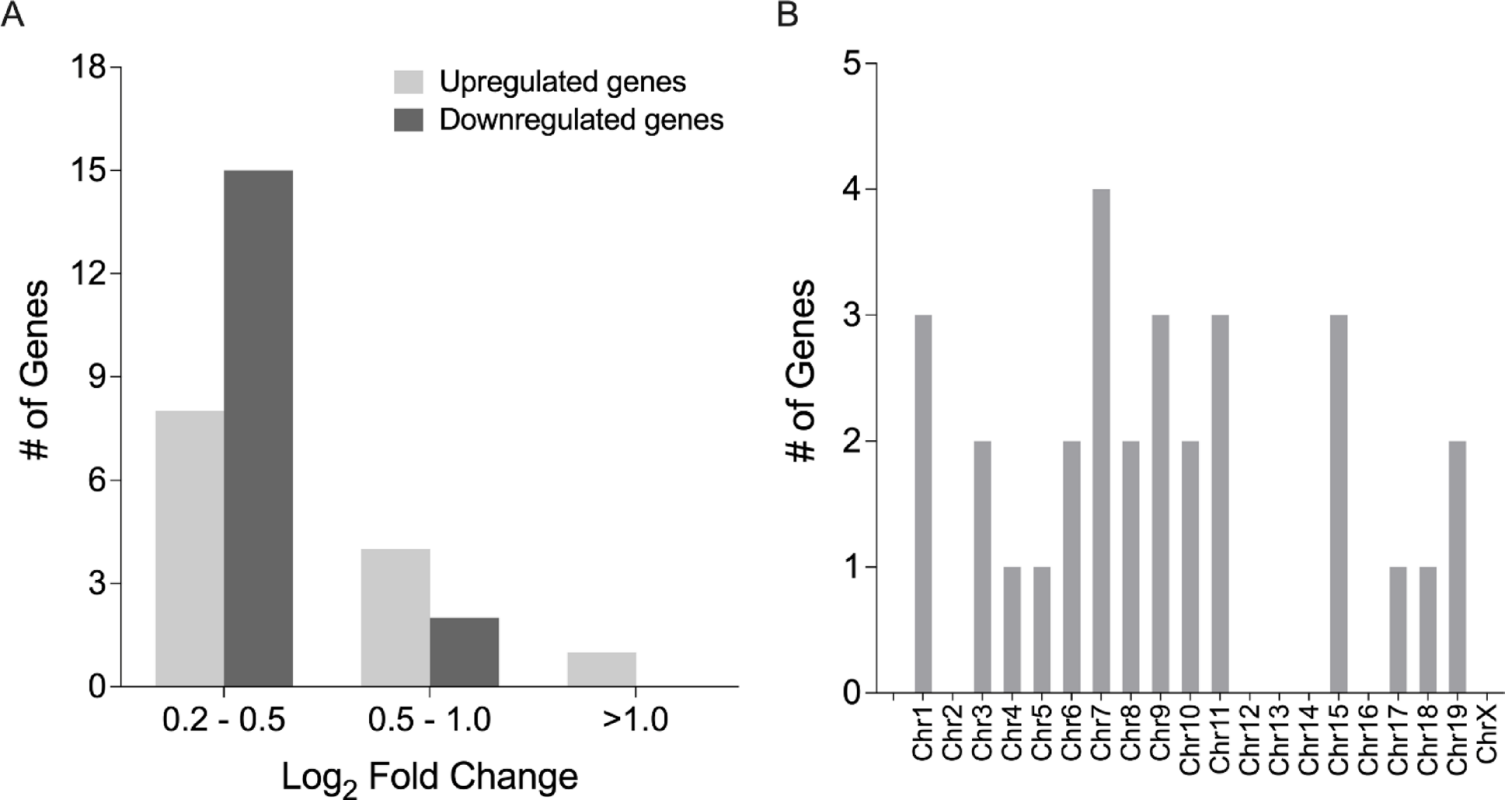
Characteristics of differentially expressed genes following OVX. (***A***) Distribution of differentially expressed genes ranked by log_2_ fold change, with most genes falling within the range of 0.2–1.0. (***B***) Distribution of differentially expressed genes across chromosomes

We used the same criteria, an adjusted *p*-value < 0.05 and a log_2_ fold change > 0.2, to define OVX-affected genes. This analysis identified 30 differentially expressed genes (Supplementary Table 5), representing a small subset of all detectable genes in the cochlea. Among these genes, 13 were upregulated and 17 were downregulated in the OVX group. Similar to the estrous cycle effect, OVX-induced expression changes were mild (Fig. 6A), with only one gene (*Gfap*) exhibiting a log_2_ fold change > 1.0. *Gfap* encodes a glial fibrillary acidic protein (GFAP), an intermediate filament protein with multiple functions, including cell-cell communication and maintenance of the blood-brain barrier in the central nervous system [72]. All identified genes were protein-coding and were located on autosomes (Fig. 6B).

#### Functional implications of OVX-affected genes

To explore the functional implications of OVX-modulated genes, we performed a DAVID analysis on the 30 differentially expressed genes identified in this study. Using the Benjamini-adjusted threshold of *p* < 0.05, as applied in the preceding DAVID analyses, no biological processes were identified (all Benjamini-adjusted *p*-values > 0.15). When the threshold was relaxed to a nominal *p* < 0.05, two biological processes were revealed: autophagy and inflammatory responses (Table 4 and Supplementary Table 3). The cellular component associated with these processes was the nucleus; however, this association did not meet statistical significance (*p* = 0.089). Further GO term analysis revealed seven enriched terms, primarily related to cell death, cell cycle, and proliferation. Although the term “inflammatory response” was also identified, its *p*-value of 0.056 exceeded our significance cutoff and was therefore excluded from the table.

**Table 4:** Biological processes and GO terms associated with OVX-modulated genes

Biological Processes	*p*-value	Gene Count	
*Functional Annotations*			
Autophagy	0.03090		3
Inflammatory response	0.04550		3
*Biological Processes*			
Positive regulation of DNA-templated transcription	0.00134		6
Autophagy	0.00183		4
Apoptotic process	0.00554		5
Regulation of cell cycle	0.02860		3
Positive regulation of glial cell proliferation	0.03120		2
Negative regulation of apoptotic process	0.03190		4
Positive regulation of epidermal growth factor receptor signaling pathway	0.03220		2

To capture the distinct functional implications of upregulated and downregulated genes, we performed GO term analysis separately for each set of genes. As shown in Table 5 (also Supplementary Table 3), downregulated genes were enriched for positive regulation of apoptotic process, DNA-templated transcription, and autophagy. In contrast, upregulated genes were associated with positive regulation of epidermal growth factor receptor signaling pathway, gene expression, SCF-dependent proteasomal ubiquitin-dependent protein catabolic process, and G1/S transition of mitotic cell cycle. These findings suggest that hormone depletion influences distinct functional pathways through different sets of upregulated and downregulated genes.

**Table 5:** GO terms associated with down and up-regulated genes after OVX

GO Term	*p*-value	Gene Count
***Down-regulated genes after OVX***		
Apoptotic process	0.00621	4
Positive regulation of DNA-templated transcription	0.00941	4
Regulation of autophagy	0.03610	2
***Up-regulated genes after OVX***		
Positive regulation of epidermal growth factor receptor signaling pathway	0.01390	2
Gene expression	0.01570	3
SCF-dependent proteasomal ubiquitin-dependent protein catabolic process	0.02900	2
G1/S transition of mitotic cell cycle	0.03730	2

Next, we performed GSEA using the Hallmark gene set collection from MSigDB, following the same approach used in Experiment 1. A FWER q-value < 0.05 was again applied as the threshold for significance. This analysis identified four gene sets associated with downregulated genes (Table 6). The top three, ranked by normalized enrichment score, were interferon-alpha response, interferon-gamma response, and oxidative phosphorylation. The enrichment of immune-related functions is consistent with results from Experiment 1, which also showed immune-related enrichment in estrous cycle-regulated genes. Additionally, we found that angiogenesis was enriched for upregulated genes, suggesting that OVX may also activate vascular growth pathways.

**Table 6:** Gene sets associated with OVX-modulated genes identified using GSEA

	Gene Set	NES	FWER q-value
Downregulated genes	Interferon alpha response	-2.54	0.000
	Oxidative phosphorylation	-2.51	0.000
	Interferon gamma response	-2.08	0.000
Upregulated genes	Angiogenesis	1.71	0.028

NES = Normalized enrichment scores; FWER = Family-wise error rate

#### Estrogen reverses OVX-induced gene expression changes

**Fig. 7:**
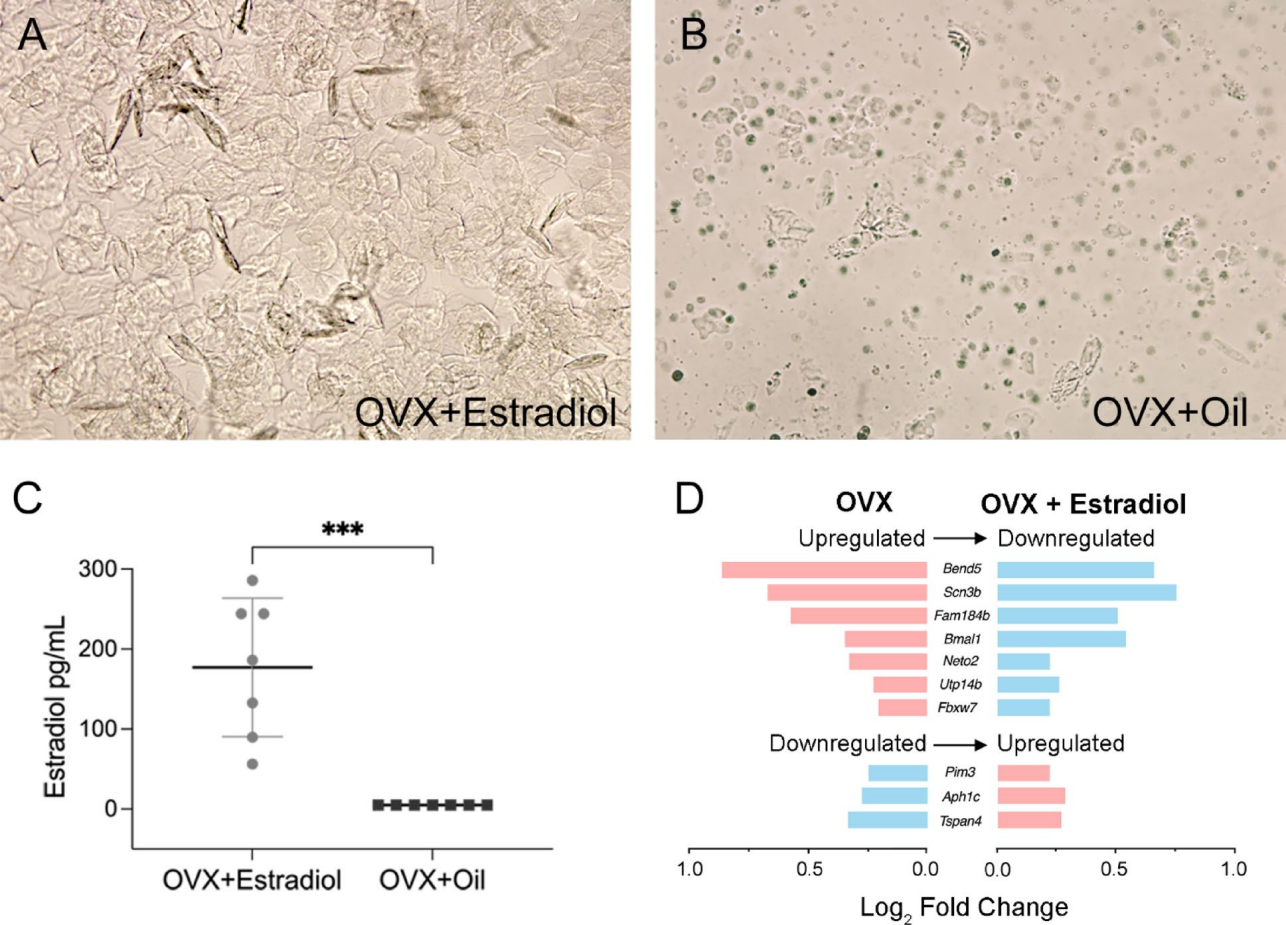
Vaginal cytology, serum estradiol levels, and gene expression changes following estradiol treatment in OVX mice. (***A***) Representative vaginal cytology image from an estradiol-treated mouse showing numerous anucleated, cornified epithelial cells and keratin bars, consistent with the estrus phase. (***B***) Representative vaginal cytology image from an untreated OVX mouse showing abundant small leukocytes and a few scattered nucleated or cornified epithelial cells, reflecting estrogen deficiency. (***C***) Comparison of serum estradiol levels between estradiol-treated and untreated OVX mice. Treated mice exhibit significantly higher serum estradiol levels than untreated mice (*** *p* < 0.001). (***D***) Changes in gene expression following estradiol treatment in OVX mice. Log₂ fold changes for the OVX group were calculated relative to sham-operated controls, and log₂ fold changes for the OVX + estradiol group were calculated relative to untreated OVX mice (OVX + sesame oil). Of the 30 genes altered by OVX, 10 responded to estradiol treatment, and all 10 exhibited reversed expression patterns. The seven genes upregulated by OVX were downregulated after estradiol treatment, whereas the three genes downregulated by OVX were upregulated following treatment

The ovary produces several gonadal hormones, including estrogen, progesterone, and androgens. Ovariectomy, therefore, can lead to reduced levels of these hormones. To identify OVX-affected genes responsive to estrogen, we treated OVX mice with estradiol and examined which genes showed reversed expression following treatment. We first examined the effect of estradiol treatment on the vaginal smear characteristics. As shown in Fig. 7A, estradiol-treated mice displayed anucleated, cornified epithelial cells, consistent with the estrus phase. In contrast, untreated OVX mice showed vaginal cytology dominated by leukocytes with a few scattered nucleated or cornified epithelial cells, indicating hormonal depletion and absence of estrous cycling (Fig. 7B). We also measured serum estradiol levels in the treated mice. As shown in Fig. 7C, the serum level of estradiol was significantly elevated in the estradiol-treated group (unpaired two-tailed Student’s *t* test, *p* < 0.001), confirming the effectiveness of estradiol administration.

We then examined RNA-seq data from estradiol-treated mice to identify changes in cochlear gene expression, applying the same criteria used previously: adjusted *p*-value < 0.05 and log_2_ fold change > 0.2. Of the 30 genes affected by OVX, 10 were modulated by estradiol treatment (Fig. 7D). Notably, all 10 genes showed expression changes in the opposite direction to those induced by OVX. Specifically, genes downregulated by OVX demonstrated increased expression following estrogen treatment, while genes upregulated by OVX were downregulated. These findings suggest that estrogen functions as a regulatory agent capable of reversing the gene expression changes in the cochlea.

## Discussion

This study investigated how sex hormones influence cochlear gene expression, with a particular focus on identifying genes responsive to female hormones. Our findings revealed a set of cochlear genes whose expression varies across the estrous cycle, most of which were autosomal and protein-coding. Diestrus-biased genes were more abundant than proestrus-biased genes. Further bioinformatics analyses revealed that many of these cycle-regulated genes have been previously identified as female hormone-responsive and are involved in various biological functions, with immune-related functions being the most prominent. Notably, enhanced expression of immune-related genes occurred at the diestrus phase. We also found that disruption of gonadal hormone production by ovariectomy altered cochlear gene expression, and that estradiol treatment reversed changes in a subset of these genes, indicating their responsiveness to estradiol. Together, these findings provide insight into how hormone-driven transcriptional changes may contribute to sex differences in auditory physiology and vulnerability to inner ear disorders.

Previous studies have documented sex differences in auditory physiology in mice, including variations in ABR wave amplitudes and susceptibility to age-related hearing loss. In C57BL/6J mice, females exhibit larger ABR wave I amplitudes than males [73], a difference that may be mediated by Gria3 expression and enhanced spiral ganglion neuron synchrony in females [74]. Additionally, female C57BL/6J mice develop high-frequency hearing loss earlier than males, with threshold elevations emerging around middle age [75–77]. In contrast, in CBA mice, males demonstrate a more rapid decline in outer hair cell function, reflected by reduced DPOAE amplitudes, along with greater reductions in peripheral auditory sensitivity, as indicated by decreased ABR amplitudes, compared with females [78]. These DPOAE declines are particularly pronounced in middle-aged male CBA mice. Collectively, these findings provide functional evidence that biological sex significantly influences auditory physiology in mice and highlight the importance of investigating hormone-associated molecular mechanisms within the cochlea.

Our current study identified 51 differentially expressed genes in the C57BL/6J mouse cochlea whose expression varied between the proestrus and diestrus phases of the estrous cycle. Functional analysis of these genes revealed association with diverse biological processes and cellular functions, with immune-related functions being the most prominent. Pathway analysis further demonstrated that these immune-related genes are enriched in the nucleotide-binding and oligomerization domain (NOD)-like receptor signaling pathway. NOD-like receptors are pattern-recognition receptors that initiate innate immune responses by detecting damage-associated molecular patterns [79]. These findings suggest that immune activity is a key target of female hormone-regulated genes in the cochlea.

Hormonal regulation of immune function during the menstrual cycle has been previously reported in clinical studies [80–82]. In females, sex hormones, including estradiol and progesterone, affect the distribution of immune cells and cytokine expression within the reproductive tract [83]. Hormonal fluctuations during the menstrual cycle also impact the clinical course of many immune-related disorders [84], including rheumatoid arthritis, multiple sclerosis, and systemic lupus erythematosus. Notably, many of these immune-related disorders have been associated with auditory dysfunction [85, 86]. Furthermore, a multi-tissue transcriptomic analysis in female mice revealed immuno-regulatory roles of the estrous cycle [87]. Our current findings, which demonstrate that the estrous cycle affects the expression of immune-related genes in female mouse cochleae, are consistent with previous studies conducted in other organs and tissues [88–90].

The menstrual cycle in humans and the estrous cycle in rodents each consist of four phases: menstrual, follicular, ovulatory, and luteal phases in humans, and proestrus, estrus, metestrus, and diestrus in rodents. Each phase is characterized by distinct hormonal patterns and physiological changes. The relationship between specific stages of the menstrual cycle and immune function has been investigated. However, the findings remain highly variable and often depend on the particular immune markers or conditions examined. For example, one clinical study reported fluctuations in 53 cytokines across the menstrual cycle, with most showing higher levels during the follicular phase compared to the luteal phase [80]. Another study found increased TNFα secretion during the luteal phase compared to the follicular phase [91]. In addition to human studies, research in mice demonstrated that immune molecule levels in the uterus and vagina changed across the estrous cycle, with some immune markers peaking during proestrus/estrus and others during diestrus [92]. Despite these discrepancies, a general pattern of female hormone effects has emerged: estrogen is associated with enhanced immune responses during the follicular and ovulatory phases, while progesterone suppresses immune activity during the luteal phase.

In our study, we found that immune-related gene expression was predominantly elevated during the diestrus phase. This raises an important question of how these changes relate to fluctuations in female hormone levels. While hormone fluctuations across the menstrual and estrous cycles are well characterized in humans and rats, reported hormone levels in mice have been more variable across studies. For example, two immunoassay-based studies reported peak estradiol levels during proestrus and peak progesterone levels during diestrus [42, 44], whereas Wood reported estradiol peaks during estrus [43]. However, a recent study by Wall et al. using liquid chromatography-mass spectrometry (LC-MS) revealed that estradiol levels peaked during diestrus, while progesterone levels peaked during proestrus [45]. These discrepancies may be attributed to differences in measurement methods, mouse strains, and the timing of sample collection. Because LC-MS is widely regarded as the most reliable method for steroid hormone analysis [93] and because Wall et al. included multiple daily sampling time points, we used their findings to guide our data interpretation. Accordingly, our finding of elevated immune-related gene expression during diestrus likely reflects increased estradiol levels and reduced progesterone levels.

It should be noted that direct comparisons of immune-related molecules across different phases of the estrous cycle between our study and previous research are challenging due to differences in the types of immune markers assessed. Our study focused on mRNA expression, whereas many previous studies examined protein expression, which do not always align with mRNA expression. Additionally, comparisons between clinical and rodent studies are further complicated by differences in cycle duration. The human menstrual cycle lasts about 28 days, whereas the mouse estrous cycle is only 4 to 5 days long, resulting in more rapid hormonal fluctuations. Such variations highlight the need for caution when interpreting and comparing findings across species.

The number of differentially expressed genes and the magnitude of gene expression changes across the estrous cycle appear to be modest in cochlear tissues. Only 51 genes were identified, with the majority showing a log₂ fold change of less than 1.0. These results suggest that the estrous cycle may have a limited impact on cochlear gene expression under physiological conditions. This contrasts with findings in the female reproductive system. For example, transcriptome analysis of the uterus across estrous cycle stages in mice identified 2,428 differentially expressed genes with a log₂ fold change greater than 1 [94]. A mild effect in the cochlea may be beneficial, helping to maintain stable gene expression despite hormonal fluctuations. However, it is also possible that the modest variation observed in this study reflects limitations of our experimental approach, which used the bulk RNA sequencing method. Because expression of female hormone receptors is cell-type specific in the cochlea [24, 95, 96], hormone-dependent changes are likely confined to specific cell populations. Therefore, the use of bulk RNA sequencing in the current study could have diluted cell-type-specific effects. Thus, our study could potentially underestimate the true magnitude of estrous cycle-dependent changes. This speculation is supported by a study of brain tissue, which found that only 5–15% of cells were hormone sensitive [66]. Yet, those cells exhibited an average of 1.9-fold change in gene expression, which is much higher than what we observed in the current study. To address this limitation, we are currently developing a single-cell RNA-seq protocol for cochlear tissue, which is expected to provide a more detailed, cell type-specific view of estrous cycle-dependent gene expression.

The current study revealed two surprising results. First, most immune-related genes whose expression varied across the estrous cycle were not detected as sex-biased in our comparison of males and females. This result differs from our initial assumption that genes regulated by female hormones would show sex-differential expression. The absence of observed sex differences may be attributed to the timing of sample collection used in our previous study [23], in which female samples were collected only in one estrous phase (the estrus phase). We predict that sampling additional stages of the estrous cycle would reveal more genes. However, such detection requires analyzing each phase separately, as merging samples across phases may increase variability and compromise gene detection. Our finding has important implications for research on sex differences. Researchers often use sex-dependent gene expression to identify genes involved in biological and disease differences between males and females. While this approach is valuable, our results suggest that it may overlook some essential genes if male-female comparisons are made without considering hormonal regulation.

The second unexpected finding was the relatively small number of differentially expressed genes identified after OVX. Only 30 cochlear genes exhibited altered expression following OVX, compared to 51 genes affected by natural hormonal fluctuations during the estrous cycle. We initially expected OVX, which presumably induces more significant hormonal changes, to produce more pronounced transcriptional alterations. This contrast prompted us to consider whether the pattern of hormonal change, sudden and sustained versus dynamic and cyclical, might differentially impact gene expression. It is possible that fluctuating hormone levels, rather than a chronic low-hormone state, may serve as stronger regulatory signals than a persistent low-hormone state. These cyclical variations may transiently modulate immune-related genes through estrogen and progesterone receptor pathways. This interpretation is supported by studies in the hippocampus and hypothalamus, where transcriptional responsiveness depends more on the timing and magnitude of estrogen surges than on baseline hormone levels [97]. Additionally, compensatory mechanisms such as extra-gonadal hormone production or local synthesis of female hormones in the cochlea may mitigate the effect of systemic hormone loss, thereby dampening transcriptional changes under OVX-induced steady-state conditions.

## Supplementary Information

Supplementary Material 1

Supplementary Material 2

Supplementary Material 3

Supplementary Material 4

Supplementary Material 5

## Data Availability

The raw RNA-seq data will be deposited in the Gene Expression Omnibus (GEO) and the Gene Expression Analysis Resource (gEAR) upon acceptance of the manuscript for publication.
